# Experience sharing on perioperative clinical management of gastric cancer patients based on the “China Robotic Gastric Cancer Surgery Guidelines”

**DOI:** 10.1186/s13741-024-00402-x

**Published:** 2024-07-25

**Authors:** Shixun Ma, Wei Fang, Leisheng Zhang, Dongdong Chen, Hongwei Tian, Yuntao Ma, Hui Cai

**Affiliations:** 1 The First School of Clinical Medicine, Lanzhou University, 1st West Donggang R.D, Lanzhou, 730000 China; 2 NHC Key Laboratory of Diagnosis and Therapy of Gastrointestinal Tumor & Key Laboratory of Molecular Diagnostics and Precision Medicine for Surgical Oncology in Gansu Province, Gansu Provincial Hospital, 204 West Donggang R.D., Lanzhou, 730000 China; 3 The Second School of Clinical Medicine, Lanzhou University, 82st Cuiyingmeng R.D, Lanzhou, 730030 China

**Keywords:** Robotic surgery, Gastric cancer, Experience sharing, China, Guidelines

## Abstract

**Background:**

With the popularization of robotic surgical systems in the field of surgery, robotic gastric cancer surgery has also been fully applied and promoted in China. The Chinese Guidelines for Robotic Gastric Cancer Surgery was published in the Chinese Journal of General Surgery in August 2021.

**Methods:**

We have made a detailed interpretation of the process of robotic gastric cancer surgery regarding the indications, contraindications, perioperative preparation, surgical steps, complication, and postoperative management based on the recommendations of China’s Guidelines for Robotic Gastric Cancer Surgery and supplemented by other surgical guidelines, consensus, and single-center experience.

**Results:**

Twenty experiences of perioperative clinical management of robotic gastric cancer surgery were described in detail.

**Conclusion:**

We hope to bring some clinical reference values to the front-line clinicians in treating robotic gastric cancer surgery.

**Trial registration:**

The guidelines were registered on the International Practice Guideline Registration Platform (http://www.guidelines-registry.cn) (registration number: IPGRP-2020CN199).

## Background

With the advancement of time and technology, robotic surgical systems have been fully applied and developed in gastric cancer surgery (Choi and Hyung 2022). China is one of the countries with high prevalence of gastric cancer worldwide and one of the countries that are currently on the leading edge in the field of robotic gastric cancer surgery (Ma et al. 2022). A total of four guidelines and consensus related to robotic gastric cancer surgery have been published in China since 2015 to date (Yu et al. 2016; Cai et al. 2021; Ma et al. 2021; Committee on Robotic and Laparoscopic Surgery of the Chinese Society of Research Hospitals et al. 2022; Upper Gastrointestinal Surgery Group of Surgical Branch Chinese Medical Doctor Association, et al. 2021), and the first Chinese guideline on robotic gastric cancer surgery was published in the Chinese Journal of General Surgery in August 2021 (Cai et al. 2021; Ma et al. 2021). Its publication marked the level of clinical treatment of robotic gastric cancer surgery in China. This guideline was jointly initiated and developed by the Minimally Invasive Surgery Committee of the Chinese Society of Research Hospitals, the Intelligent Equipment Technology Branch of the China Medical Equipment Association and Gansu Provincial People’s Hospital, with methodological support from the Center for Evidence-Based Medicine, School of Basic Medicine, Lanzhou University/World Health Organization Collaborating Center for Guideline Implementation and Knowledge Translation/Gansu Medical Guideline Industry Technology Center. The purpose is to further standardize the diagnosis and treatment criteria of robotic gastric cancer surgery, ensure medical quality, and improve clinical diagnosis and treatment. The guidelines were developed in accordance with WHO guidelines, and the quality of evidence and strength of recommendations were assessed by using the GRADE (grading of recommendations assessment, development and evaluation) method and the supplement to the GRADE grading system (good practice statement, GPS), and finally the modified Delphi method was used to reach consensus on the recommendations (Perillat and Mercuri 2022; Galvao Neto et al. 2022). The guidelines were registered on the International Practice Guideline Registration Platform (http://www.guidelines-registry.cn) (registration number: IPGRP-2020CN199). The guidelines contained recommendations on 17 clinical issues, including 14 strong recommendations and 3 weak recommendations, and a questionnaire was administered to clinical issues that may have different patient preferences and values. After more than a year of promotion and application, the guideline has been well received both nationally and internationally. In order to further dissect the guideline recommendations and improve the results of robotic gastric cancer surgery treatment. Based on the recommendations of this guideline and the problems encountered in the current clinical work, this article describes the indications and contraindications for surgery, perioperative preparation, surgical steps, complication prevention and treatment and postoperative management during robotic gastric cancer surgery, and summarizes 20 experiences of perioperative clinical management of robotic gastric cancer surgery based on other surgical guidelines, consensus, and single-center experience, hoping to give front-line clinicians a better understanding of the treatment of robotic gastric cancer. We hope to bring some clinical reference value to the front-line clinicians in the treatment of robotic gastric cancer surgery.

## Recommendations of the “Chinese Guidelines for Robotic Gastric *Cancer* Surgery”

### Indications for surgery (Recommendation 1)

① Primary gastric cancer stage I, II, III (except in situ cancer). ② Gastroscopy and pathological examination confirm the diagnosis of gastric cancer, without distant metastases such as liver or lung by clinical and imaging examination. ③ The patient is in good physical condition to tolerate the surgery, without serious organ insufficiency such as heart, lung, brain, liver, and kidney. ④ Exploratory surgery is feasible for gastric cancer combined with perforation, bleeding, obstruction, etc. (Ajani et al. 2022; Huang et al. 2018). (GPS).

### Contraindications to surgery (Recommendation 2)

① Extensive metastases in the abdominal cavity and other organs detected by PET or CT examination. ② With vital organ failure and poor general condition, unable to tolerate surgery. ③ Coagulation disorders. ④ Pregnancy and inability to tolerate CO_2_ pneumoperitoneum (Ajani et al. 2022; Huang et al. 2018). (GPS).

### Patient preoperative preparation and psychological counseling (Recommendation 3)

① Assess the patient’s general condition, stratify pulmonary and cardiovascular, renal and cerebrovascular risks, and evaluate any contraindications to surgery. ② Improve the patient’s general condition, actively and effectively manage comorbidities that may increase the risk of surgery or affect the efficacy, and exercise cardiopulmonary function. ③ Evaluate the nutritional status and correct disorders of water-electrolyte acid–base balance, anemia, and hypoproteinemia. ④ Eat liquid food 1 day before surgery, fast for 8 and 4 h on the day of surgery, if the fasting time is too long, infuse glucose sodium chloride solution. If the fasting time is too long, glucose sodium chloride solution can be infused. ⑤ In case of combined colectomy, intestinal preparation (oral laxative or enema) 1–3 days before surgery. ⑥ Prophylactic antibiotics 30 min before surgery (Zheng et al. 2016; Corcione et al. 2018). ⑦ In-depth communication with the patient, detailed explanation of the surgical plan and introduction of the surgical specialist, to relieve the patient’s concerns about the effect of robotic surgery. ⑧ Eliminate patients’ negative attitudes and improve sleep quality (Yan et al. 2017). (GPS).

### Medical and nursing preparation (Recommendation 4)

① Preoperative tumor assessment, comprehensive determination of tumor location, scope and TNM stage through gastroscopy or ultrasound gastroscopy, enhanced CT, MRI, upper gastrointestinal imaging and other examinations. ② Determine the treatment strategy and standardized whole treatment plan through multidisciplinary treatment (MDT) mode discussion. ③ Sign the informed consent for surgery and explain the specifics of robotic surgery to the patient. ④ The nursing staff need to prepare the patient for skin preparation in the operation area, antibiotic skin test and enema before surgery, and inform the patient of preoperative and postoperative care precautions (Corcione et al. 2018; Professional Committee of Robotic and Laparoscopic Surgery in Chinese Society of Research Hospitals, et al 2020; Ji et al. 2016). (GPS).

### Preparation of surgical equipment and instruments (Recommendation 5)

① Surgical Equipment Preparation: Robotic surgical system, laparoscopic high-definition camera display system or 3D camera display system, automatic high-flow pneumoperitoneum machine, suction and irrigation machine, ultrasonic knife and electrocoagulation system, video and image storage equipment, etc. Before use, it is necessary to turn on the robot system, check the instruments, especially whether the robotic arm can be used normally, install a special disposable sterile sleeve for the robotic arm, connect the robotic observation lens light source, and after focusing and three-dimensional calibration, heat the lens to prevent fogging. ② Surgical instrument preparation: pneumoperitoneum needle, puncture device, conversion cannula, non-invasive surgical grasping forceps with bipolar electrocoagulation and Maryland grasping forceps, cross calibrator, applicator and hemostatic clamp, large needle holder, monopolar electric scissors, electric hook, ultrasonic knife, incisional closure, anastomosis, intracavitary linear cutting closure, disposable pick-up bag, special supporting instruments for the use of the instrument arm, etc. ③ Other preparations: The operator can choose robotic and laparoscopic instruments to be used in conjunction with each other according to the hospital’s own conditions and personal habits (Committee for the Development of the Guidelines for Robotic Hepatobiliary and Pancreatic Surgery Operation 2019). (GPS).

### Choice of surgical approach and type (Recommendation 6)

① The recommended surgical approaches were fully robotic and robotic-assisted, and their differences were not statistically significant in terms of patient survival, but the incision length was smaller in the fully robotic group. (recommended, very low quality) ② The recommended types of surgery were proximal gastrectomy, distal gastrectomy, pylorus-preserving gastrectomy, and total gastrectomy. In distal and total gastrectomy, robotic surgery can obtain a higher number of lymph node dissections with fewer complications (Luo et al. 2019; Kuang et al. 2020; Chen et al. 2017). (recommended, very low quality).

### Selection of surgical anesthesia (Recommendation 7)

General anesthesia with endotracheal intubation is recommended, and general anesthesia combined with epidural anesthesia can also be used (Yu et al. 2016). (GPS).

### Surgical position and poke card layout selection (Recommendation 8)

① The recommended surgical position is with the patient in a supine position with legs parted, head high and feet low at 15–30°, with an appropriate tilt to the right side at 10–20°. (GPS) ② Recommended poke card layout pattern (using da Vinci si machine as an example). In the general mode W-shaped 5-hole layout is adopted, with 2 cm below the umbilicus as the observation hole and establishment of pneumoperitoneum, the left anterior axillary line under the rib cage as the 1st operation hole, the left midclavicular line 2 cm below the flat umbilicus with a 12-mm poke card as the assistant hole, the right anterior axillary line under the rib cage as the 3rd operation hole, and the right midclavicular line 2 cm below the flat umbilicus as the 2nd operation hole, with the distance between adjacent poke cards greater than 8 cm to avoid mutual interference of mechanical arms. In the “3 + 2” mode, a 5-mm poke card is placed in the 3rd operation hole as the 2nd assistant hole, and the rest of the poke cards are laid out in the same way as the general mode (Yu et al. 2016; Zheng et al. 2016; Hu et al. 2019). (GPS).

### Precautions during abdominal entry and exploration (Recommendation 9)

Establish pneumoperitoneum with pneumoperitoneum pressure of 10–12 mmHg (1 mmHg = 0.133 kPa), firstly explore the abdominal cavity, then investigate the liver, diaphragm, mesentery, peritoneum, pelvis, and ascites in order from near to far, determine the tumor site, size and surrounding lymph nodes, and then make sure that robotic radical gastric cancer surgery can be performed. The robotic arm is then installed and fixed. At present, there are three main approaches commonly used in robotic gastric cancer surgery: left posterior approach, left anterior approach, and right anterior approach. It can be applied flexibly according to the experience of the surgical team, the tumor condition, the patient’s body type, and the choice of the digestive tract reconstruction modality (Yu et al. 2016; Committee on Robotic and Laparoscopic Surgery of the Chinese Society of Research Hospitals, et al.^2021^). (GPS).

### Selection of the extent of surgical resection (Recommendation 10)

① The scope of surgical resection is divided according to the tumor site, and it is recommended to perform proximal gastrectomy for combined esophagogastric cancer, which has no statistically significant difference from total gastrectomy in terms of anastomotic leak, reflux, and intestinal obstruction. (recommended, very low quality) ② Proximal gastrectomy for upper 1/3 gastric cancer is recommended to achieve long-term survival similar to that of total gastrectomy, while proximal gastrectomy reduces the incidence of postoperative intestinal obstruction and abdominal abscesses. (recommended, low quality) ③ Distal gastrectomy is recommended for middle and lower 1/3 gastric cancers, with better long-term prognostic performance than total gastrectomy, and distal gastrectomy may also reduce postoperative related complications (Chen et al. 2019; Wang et al. 2020a, 2018; Dang et al. 2020; Zhao et al. 2021; Li et al. 2018). (Recommended, low quality).

### Scope of intraoperative lymph node dissection and precautions (Recommendation 11)

① Scope of intraoperative lymph node dissection: Proximal gastrectomy with lymph nodes of D1 + No.8a, No.9 and No.11p groups. Distal gastrectomy to remove lymph nodes of D1 + No.8a, No.9, No.11p, No.12a group. Pylorus preserving gastrectomy (PPG) Lymph nodes of D1 + No.8a, No.9 group were cleared. Total gastrectomy to remove lymph nodes of D1 + No.8a, No.9, No.10, No.11p, No.11d, No.12a. (No.8p, No.12b, No.12p group lymph nodes can be selectively dissected according to the physician and patient’s specific situation). (GPS) ② Intraoperative considerations: During lymph node dissection, the whole block resection method is recommended, while paying attention to the protection of important blood vessels and other organs, and not damaging the vascular sheath as much as possible. During the lymph node dissection of No.8p, No.12a group, thicker lymphatic vessels should be clamped to prevent postoperative lymphatic leakage (Yu et al. 2016; Bobo et al. 2019; Japanese Gastric Cancer Association 2022; Hu and Zhang 2018; Gastric Cancer Professional Committee of China Anti-Cancer Association 2020). (GPS).

### Intraoperative management of resected specimens and choice of GI reconstruction modality (Recommendation 12)

① Intraoperative management of resected specimens: For distal gastric and pylorus-preserving gastrectomy, it is recommended that the specimen is first removed completely under full robotics, the cut edge is inspected and then loaded into a specimen bag, the surgical area is flushed, anastomosis is reconstructed, and finally the specimen bag is removed through an auxiliary incision. For proximal gastrectomy and total gastrectomy, it is recommended to free the specimen intact under the full robot, perform anastomotic reconstruction with the specimen removed, inspect the margins, put the specimen in a pouch, rinse the surgical area, and finally remove the pouch through an auxiliary incision. It is also possible to free the specimen intact first, remove it after dragging it out through the auxiliary incision, inspect the incision margins, and perform open anastomosis reconstruction or temporarily close the auxiliary incision for anastomosis reconstruction under the robot. (GPS) ② Choice of digestive tract reconstruction modality: Gastrointestinal reconstruction for proximal gastric cancer radical surgery (D2 radical surgery) can be performed by esophagogastric anastomosis, which is superior to interposition jejunostomy in terms of hospitalization time, operative time, and intraoperative bleeding. The incidence of anastomotic stricture is less with dual-channel anastomosis than with esophagogastric anastomosis. There was no statistically significant difference between interposition jejunostomy and dual-channel anastomosis in terms of postoperative anastomotic stricture and anastomotic fistula. For distal gastric cancer radical reconstruction (D2 radical surgery), Billroth I anastomosis can be used, which reduces the overall complication rate compared to Billroth II and Roux-en-Y anastomoses. There was no statistically significant difference in the overall complication rate between Billroth II and Roux-en-Y anastomoses. Total gastric cancer radical gastrointestinal reconstruction (D2 radical surgery) with an esophago-jejunal Roux-en-Y anastomosis, which allows the use of an intestinal pouch as a gastric substitute, with a storage pouch Roux-en-Y anastomosis reduces postoperative dumping syndrome and decreases the overall postoperative complication rate, with no statistically significant difference between operative time and Roux-en-Y anastomosis without a storage pouch (Shaibu et al. 2020; Tanaka et al. 2020, 2019; Kim et al. 2019; Kimura et al. 2020; Kinoshita et al. 2020; Nakanishi et al. 2020; Syn et al. 2019; Chen et al. 2018). (Recommended, very low quality).

### Intraoperative complications and management (Recommendation 13)

① Intraoperative complications: pneumoperitoneum, intraoperative vascular injury, intraoperative adjacent organ injury, etc. (Yu et al. 2016; Committee on Robotic and Laparoscopic Surgery of the Chinese Society of Research Hospitals, et al. 2021). ② Intraoperative management: For pneumoperitoneum-related complications, the pneumoperitoneum pressure should be closely monitored to avoid subcutaneous emphysema, keep the muscles in a good relaxed state during surgery, and shorten the operation time as much as possible. Intraoperative vascular injury, the normal anatomical position of the perigastric vessels should be mastered, the variation of the intraoperative vascular position should be noted, the correct surgical plane should be revealed, once bleeding occurs, the correct hemostatic tools should be used, and if bleeding is difficult to control, intermediate laparotomy should be considered. For intraoperative adjacent organ injury, the operator should master the anatomical position and structure of the adjacent organs around the stomach, choose the appropriate access, and perform the separation operation along the correct surgical plane (Aktas et al. 2020; Hikage et al. 2021; Kong et al. 2020; Roh et al. 2020a; Tian et al. 2022; Yang et al. 2020a; Chen et al. 2020; Cui et al. 2020; Liao et al. 2020; Liu et al. 2020; Qin et al. 2020). (GPS).

### Postoperative complications and management (Recommendation 14)

① Postoperative complications: anastomotic bleeding, anastomotic leak, duodenal stump leak, pancreatic leak, intestinal obstruction, etc. (Yu et al. 2016; Committee on Robotic and Laparoscopic Surgery of the Chinese Society of Research Hospitals, et al. 2021;). ② Postoperative management: Routine postoperative anastomotic bleeding can be treated conservatively. When the bleeding volume is greater than 100 ml/h, endoscopic or open surgery should be performed immediately to stop the bleeding. Once an anastomotic leak occurs, it should be given promptly with patulous drainage, and reoperation is required if necessary. In case of duodenal stump leak, abdominal drainage and parenteral nutritional support should be provided. For patients whose conservative treatment is ineffective or combined with serious complications, surgical treatment should be given. In case of pancreatic leakage, abdominal double trocar flushing and drainage and suppression of pancreatic exocrine secretion should be performed, and surgical drainage and lavage should be given if necessary. If intestinal obstruction occurs after surgery and conservative treatment is ineffective, surgical treatment should be given (Aoyama et al. 2012; Jex et al. 1987; Makuuchi et al. 2019; Orsenigo et al. 2014; El-Sayes et al. 2017; Tharavej et al. 2019). (GPS).

### Postoperative patient management considerations (Recommendation 15)

① Closely observe the condition, give cardiac monitoring, oxygen, observe the patient’s mental and vital signs, and record the in and out volume. ② Care for the incision and drainage tube, closely observe the incision and ensure that the drainage tube is in place. ③ Early feeding, after the anus is exhausted, the patient can eat liquid food, and gradually transition to diet according to the patient’s specific situation. ④ Encourage patients to get out of bed at an early stage. ⑤ Postoperative pulmonary function protection, postoperative nebulized inhalation to dissolve sputum, back patting or mechanical sputum excretion. ⑥ Postoperative coagulation management, hemostatic drugs can be given on the same day after surgery, and coagulation function should be tested regularly after surgery and low-molecular heparin calcium should be given. ⑦ Postoperative nutritional support, postoperative parenteral nutrition, or enteral nutrition support. ⑧ Postoperative analgesic treatment, postoperative analgesic pump, or analgesic injection is given for analgesia (Lemmens et al. 2009; Bi et al. 2013; Ji 2013; Choi et al. 2020; Wang and Pan 2011; Roh et al. 2020b; Xue et al. 2021). (GPS).

### Postoperative specimen retrieval and delivery method (Recommendation 16)

The surgical resection specimen of robotic gastric cancer can be directly removed through the median upper abdomen or subumbilical incision before or after the completion of GI reconstruction. The surgical specimen should be removed in a disposable specimen bag, and the abdominal cavity and incisional implantation metastasis should be avoided during the removal process. In addition, fine sorting of the perigastric lymph nodes of each group should be performed immediately after specimen isolation to obtain sufficient lymph nodes and thus accurate information on lymph node metastasis. They should be fixed with 10% formaldehyde solution within 30 min and sent to the pathology department for routine immunohistochemistry and detection of common gastric cancer molecular targets (Deng 2019; Yuan and Ma 2018). (GPS).

### Accelerated rehabilitation program (Recommendation 17)

Accelerated rehabilitation surgical care is recommended to shorten the patient’s hospital stay and promote rapid anal venting, while reducing hospital costs (Xue et al. 2021; Zeng et al. 2019; Tang et al. 2020; Wang and Wang 2018; Committee of Robotics and Laparoscopic Surgery in Chinese Society of Research Hospitals 2016; Mortensen et al. 2014; World Health Organization 2014; Jiang et al. 2016). (Recommended use, low quality).

## Experience in perioperative management of patients with gastric cancer

### Experience in the management of patients with super-indications

① Locally advanced or with distant metastases: Neoadjuvant chemotherapy can be chosen for downstaging and then re-evaluated to decide whether to choose to continue surgery. The recommended chemotherapy regimen is FLOT with 3–6 cycles of chemotherapy. Combination immunotherapy and targeted therapy can also be selected according to the results of immunohistochemistry and genetic testing (Grizzi et al. 2022).

② Gastric cancer combined with perforation: Radical resection can be attempted for gastric cancer combined with perforation after stable condition through conservative treatment. Pneumoperitoneum can be established first for laparoscopy, and if the condition is suitable for minimally invasive surgery, robotic surgery can be continued; otherwise, open abdomen can be performed. If the tumor can be completely resected, total or distal gastrectomy is feasible, and whether lymph node dissection should be performed is decided according to the specific situation. Postoperative placement of jejunal nutrition tube and gastrointestinal decompression tube is recommended to prevent anastomotic leakage and facilitate postoperative nutritional support, placement of abdominal drainage tube and/or double trocar for unobstructed drainage, and flushing of abdominal cavity with plenty of distilled water. If the tumor cannot be completely resected, repair of the perforation with a large omentum, gastrostomy, and jejunostomy, and placement of an abdominal drain and double trocar for patency drainage are recommended (Itoh et al. 2022).

③ Gastric cancer combined with bleeding: It is recommended to place a gastrointestinal decompression tube to observe the bleeding, and if the bleeding is not large, oral Yunnan Baiyao powder and aluminum-magnesium plus suspension or norepinephrine ice saline can be administered to stop the bleeding, and intravenous or intramuscular hemostatic drugs can be given simultaneously or successively, and endoscopy and local sclerosis to stop the bleeding are also feasible. If the bleeding stops after conservative treatment, the patient’s status should be re-evaluated, and if surgery is needed, the patient should be fully prepared for robotic gastric cancer surgery by the deadline, and if surgery is not possible, the patient should be evaluated whether chemotherapy or other treatments are needed. If the bleeding has not stopped after conservative treatment, and the patient’s vital signs, hemoglobin, and platelets can tolerate the basic requirements of general anesthesia surgery, pneumoperitoneum can be established and laparotomy can be performed first, and if the conditions are suitable for minimally invasive surgery, robotic surgery can be continued; otherwise, open surgery can be performed. Postoperatively, it is recommended to place jejunal nutrition tube and gastrointestinal decompression tube to prevent anastomotic leakage and facilitate postoperative nutritional support, and to place abdominal drainage tube for smooth drainage. If the tumor cannot be completely resected or if the condition turns rapidly, ligation of the left gastric artery and right gastric artery is recommended. If the patient’s status cannot tolerate general anesthesia, it is recommended to give gastric vascular embolization through vascular intervention to control bleeding and transfer to ICU for advanced life support, and then decide whether to perform surgical resection after the patient’s status has recovered or corrected (Yagi et al. 2022).

④ Gastric cancer combined with pyloric obstruction: In this case, placement of a gastrointestinal decompression tube to aspirate gastric contents is recommended. Before placement, it is recommended to trim and enlarge the lateral orifice of the gastric tube to improve the suction effect, and slow suction is recommended when there is a large amount of retained material in the stomach to prevent the occurrence of gastric mucosal bleeding due to too fast suction. It is also recommended to drink light saline to rinse the gastric cavity to reduce the edema of the gastric wall, and to take oral gentamicin or levofloxacin for tumor necrosis infection. Assess the tumor stage and perform robotic distal gastrectomy if radical resection is possible; if not, invite MDT consultation to assess whether neoadjuvant chemotherapy, stent placement, or gastrojejunostomy is needed. For gastric cancer with cardia obstruction, a nasogastric tube is recommended to aspirate the esophageal contents, assess the tumor stage, and perform robotic total gastrectomy or proximal gastrectomy if radical resection is possible; if not, MDT consultation is invited to assess whether gastrojejunostomy, neoadjuvant chemotherapy, radiotherapy, or esophageal stenting is needed. In case of incomplete obstruction, enteral nutrition support can be given through gastroscopic placement of a gastrojejunal nutrition tube. In complete obstruction, gastrostomy or jejunostomy for enteral nutritional support is possible (Jiao et al. 2021).

### Experience in the exclusion of contraindications to surgery

Robotic gastric cancer surgery is not recommended for patients who meet Recommendation 2. The patient’s physical condition was assessed, and MDT consultation was invited to discuss whether other treatments such as chemotherapy and radiotherapy could be tolerated. If any anti-tumor treatment is not tolerated, nutritional support and analgesic treatment are given to provide good clinical care (Xiang et al. 2022).

### Patient preoperative preparation experience

Patients should be adequately prepared preoperatively for robotic gastric cancer surgery on a deadline according to the requirements of Recommendation 3. In order to prevent patients’ lower extremity deep vein thrombosis, preoperative ultrasound examination of both lower extremity veins is recommended, together with DVT risk factor scale (Kaprini scale) scoring, and elastic stockings should be worn in advance on the patient’s surgery day. Long-term use of antiplatelet and anticoagulant drugs such as aspirin, forest clopidogrel, and warfarin is recommended to be stopped 1 week in advance and replaced with low-molecular heparin. Preoperative monitoring of cardiac enzymes and myocardial markers and examination of ambulatory 24-h electrocardiogram are recommended. If necessary, coronary CTA is performed, or coronary angiography under intervention is performed. Cardiology consultation is invited to assist in treatment. Hypertensive patients can continue to take antihypertensive medication on the day of surgery. Antihypertensive medication containing reserpine must be stopped for 1 week, and blood pressure is maintained within 140–160/90–100 mmHg before surgery. The 24-h ambulatory blood pressure and 24-h ambulatory electrocardiogram were measured. Invite cardiology consultation to assist in treatment. Diabetic patients should stop the use of hypoglycemic drugs or insulin injection on the day of surgery and maintain fasting blood glucose within 6–10 mmol/L and glycated hemoglobin within 6–10 mmol/L before surgery. Glucose spectrum was measured, blood glucose was monitored continuously for 24 h, and endocrinology or diabetes department was invited to consult and assist in treatment. Patients with renal insufficiency should improve renal function before surgery, and if necessary, hemodialysis should be performed 1 day before surgery to maintain blood creatinine within 100 mmol/L and blood urea nitrogen within 20 mmol/L. Patients with a history of previous cerebral infarction or cerebral hemorrhage should have a preoperative review of cranial MRI to assess changes in their condition, and surgery should be gradual and controlled more than 6 months from the most recent cerebrovascular event. Neurology consultation is invited to assist in the treatment. Patients who smoke must strictly abstain from smoking for more than 1 week before surgery and exercise their lung function. Patients who drink alcohol must strictly abstain from alcohol for more than 1 week prior to surgery and discontinue medications that impair liver function. Surgery is suspended during menstruation in female patients (Association et al. 2021).

### Medical and nursing preparation experience

The attending surgeon, anesthesiologist, and operating room nurse in charge of surgery need to visit the patient 1 day before surgery, ask about the patient’s past medical history, surgical history, allergy history, blood transfusion history, infectious disease history, evaluate the patient’s examination and laboratory results, and prepare for surgery in advance. If abnormalities are found, they should be communicated with the attending physician and dealt with in a timely manner. If the patient’s surgical risk is really high, suspend the surgery if necessary and do it at a later date. The attending physician should check the patient’s blood type, prepare for intraoperative blood transfusion in advance, send the matched blood to the transfusion department, and contact the blood station to get blood or family members to donate blood if there is no blood reserve of that type. The physician should write the preoperative visit record, preoperative summary, and preoperative discussion and sign the informed consent form for blood transfusion. For patients with early tumor stage, it is recommended to mark tumor location under electronic gastroscopy 1 day before, on the same day or intraoperatively to do accurate tumor localization, which can be done by local injection of methylene blue, fluorescein or nano carbon, or by endoscopic hemostatic clips, and also by intraoperative ultrasound. Intraoperative lymph node identification can be performed by local injection of nanocarbon or indole turnip green (Felsenreich et al. 2020).

### Experience in preparation of surgical equipment and instruments

In addition to the equipment that needs to be prepared routinely for robotics, electronic gastroscopy equipment or intraoperative ultrasound machines need to be prepared in advance if intraoperative gastroscopy or intraoperative ultrasound is to be performed for the patient. Prepare various types of surgical sutures commonly used in gastrointestinal surgery (Ning et al. 2017).

### Experience with surgical modality and type selection

Surgical modalities are divided into fully robotic and robotic-assisted, and robotic-assisted surgery is recommended for operators with skilled robotic surgery skills feasible for fully robotic surgery. Patients with high BMI, difficult surgery, and average surgical skills are recommended for robotic-assisted surgery. The types of surgery are classified as proximal gastrectomy, distal gastrectomy, pylorus preserving gastrectomy, and total gastrectomy according to the location, size, and boundary of tumor growth (Committee on Robotic and Laparoscopic Surgery of the Chinese Society of Research Hospitals, et al. 2021).

### Patient anesthesia management experience

General anesthesia is mostly used clinically, and deep venous and arterial puncture tubes are given to facilitate intraoperative fluid rehydration and dynamic monitoring of blood pressure and arterial blood gases. Blood gas testing should be performed before, during, and at the end of surgery, and arterial blood gas can be tested every hour if the operation is prolonged. Observe changes in PH, lactate, hemoglobin, electrolytes, oxygen saturation, and other indicators, and deal with any abnormal changes in a timely manner. Pay attention to maintain the depth of anesthesia during surgery, keep blood pressure and heart rate stable, use more short-acting anesthetics for general anesthesia induction and general anesthesia maintenance, and choose the concentration of inhaled anesthetic drugs carefully. Pressure control ventilation, reduce airway pressure, and increase oxygenation. Prolong the inspiratory-to-expiratory time ratio, improve the functional residual air volume, prevent alveolar atrophy, and reduce intrapulmonary shunts. Apply heating blankets and warmers to keep the patient warm intraoperatively. Appropriate additional inotropic drugs were used intraoperatively to keep the abdominal muscles relaxed and maintain the pneumoperitoneum space. Intraoperative gastric and intestinal wall edema and exudate can be given infusion of human albumin to improve plasma osmolarity. Transversus abdominis fascial block, lumbar square muscle block, or incisional block or local application of lidocaine gel can be given during closure, and analgesic pump can be given for postoperative analgesia (Zhang et al. 2021).

### Experience in selecting surgical body position and poke card layout

The fourth-generation da Vinci Surgical Robot System Xi comes with a supporting surgical bed, which can automatically adjust the body position and locate the operation hole position automatically after infrared scanning and positioning through the surgical site. The third-generation da Vinci Surgical Robot System Si requires manual operation of the surgical bed to adjust the patient’s position. The supine position is often used, with the head high and foot low 15–30° and the right side tilted 10–20°. Intraoperatively, 1–2 assistants are generally required to participate in the surgery, and the location of the operating holes is reasonably laid out according to the principles of upper abdominal surgery operation, with adjacent operating holes spaced > 8 cm apart as much as possible to avoid mutual interference (Ong et al. 2022).

### Precautions for abdominal access and exploration

It is recommended that according to the lumpectomy procedure, after selecting the location of the lens hole, the skin is incised, and the length of the skin incision should not exceed the diameter of the poke card as much as possible, the abdominal wall is lifted upward after clamping the skin on both sides of the incision with cloth towel clamp, and the pneumoperitoneum needle is stabbed vertically downward into the abdominal cavity through the incision, and successful puncture is considered when two popping sounds are heard and there is a sense of falling air, and the pneumoperitoneum tube is connected and inflated into the abdominal cavity. Observe the changes of pneumoperitoneum flow and pressure values, and tap the abdominal wall when the abdomen is elevated with obvious drumming sounds indicating successful inflation. Pull out the pneumoperitoneum needle, change the lens hole poke card again for puncture, when there is a feeling of falling empty, open the poke card air inlet valve if there is gas outflow to prove successful entry into the abdominal cavity. At this point, the puncture core is removed and the surgical lens is inserted from the poke card to verify successful entry into the abdominal cavity, and the depth of the poke card is adjusted to the marked scale. It is important not to blindly puncture the poke card directly before establishing the pneumoperitoneum, as this could easily accidentally injure the abdominal organs. Continue to puncture additional poke cards under direct lens vision. The pneumoperitoneum pressure was maintained at 10–12 mmHg and the flow rate was 40 L/min. When performing the abdominal exploration, we first observed whether there was any organ damage directly below the lens hole, and then explored the abdominal cavity in order from near to far, observed whether there were metastases and ascites, determined the tumor site, size and surrounding lymph nodes, and then installed and fixed the robotic arm after it was clear that robotic radical gastric cancer surgery could be performed (Siddiqi and Johnston 2023).

### Experience in selecting the extent of surgical resection

Proximal gastrectomy is feasible for patients with combined esophagogastric cancer and upper 1/3 gastric cancer, and distal gastrectomy is feasible for patients with middle and lower 1/3 gastric cancer (Jiang et al. 2022).

### Intraoperative lymph node dissection scope and precautions

The scope of lymph node dissection refers to the 5th edition of the Japanese Guidelines for the Treatment of Gastric Cancer, the 16th edition of the Japanese Statute for the Management of Gastric Cancer, the Operating Guidelines for Laparoscopic Gastric Cancer Surgery (2016 edition), and the Chinese Expert Consensus on Difficulties in the Diagnosis and Treatment of Gastric Cancer (2020 edition), and the scope of dissection can be divided into proximal, distal, and whole stomach. The principle of complete debridement, i.e., “en bloc,” is followed during surgery to avoid lymph node fragmentation (Yu et al. 2016; Japanese Gastric Cancer Association 2022; Hu and Zhang 2018; Gastric Cancer Professional Committee of China Anti-Cancer Association 2020; Gastric Cancer Association, China Anti-Cancer Association 2021; Felsenreich et al. 2020; Ning et al. 2017; Zhang et al. 2021; Ong et al. 2022; Siddiqi and Johnston 2023; Jiang et al. 2022; Zhao et al. 2020).

### Experience with specimen removal and GI reconstruction

The specimen can be completely resected before the GI anastomosis reconstruction under full robotics, or the specimen can be resected after the GI anastomosis reconstruction by the self-traction post-dissection method (SPLT), checked the resected specimen cut edge, put into the specimen bag, rinsed the surgical area, and finally removed by the auxiliary incision or NOSES method. With robotic assistance, the specimen can be freed intact, dragged out through the auxiliary incision and then resected, followed by open anastomosis reconstruction or temporary closure of the auxiliary incision for anastomosis reconstruction under the robot. After radical surgery for proximal gastric cancer, esophagogastric anastomosis, interposition jejunostomy, or dual-channel anastomosis can be used. Billroth I, Billroth II, or Roux-en-Y anastomosis can be used after radical and pylorus-preserving gastrectomy for distal gastric cancer. An esophagus-jejunum Roux-en-Y anastomosis can be used after radical total gastrectomy, and an enteric pouch can be used as a gastric substitute (Upper Gastrointestinal Surgery Group of Surgical Branch Chinese Medical Doctor Association, et al. 2021; So et al. 2018).

### Postoperative specimen delivery and lymph node sorting experience

To ensure the accuracy of postoperative pathology reports, it is recommended that specimens be handled by specialized personnel. After removing the specimen, wipe clean the blood stains and dirt, lay it flat on a clean specimen table, unfold it, and take pictures of the front and back side, and place a ruler next to it. Then the stomach was neatly cut along the contralateral edge of the tumor, wiped clean of blood stains and dirt, and photographed again. The size of the tumor and the distance between the upper and lower cut edges were measured. And fine sorting of each group of perigastric lymph nodes is performed. Lymph node sorting procedure: refer to “Chinese Expert Consensus on Standardized Surgical Treatment of Radical Gastric Cancer Specimens (2022 Edition).” ① Proximal gastrectomy should at least sort the lymph nodes of groups No.1, No.2, No.3, No.4sa, No.8a, No.9, No.11p separately; ② Distal gastrectomy should at least sort the lymph nodes of groups No.3, No.4sb, No.4d, No.5, No.6, No.8a, No.9, No.11p, No.12a separately; ③ Pylorus preserving gastrectomy (PPG) at least group No.3, No.4sb, No.4d, No.8a, No.9 lymph nodes should be sorted separately; ④ Total gastrectomy at least group No.1, No.2, No.3, No.4sa, No.4sb, No.4d, No.5, No.6, No.7, No.8a, No.9, No.11, No.12a lymph nodes should be sorted separately. No.12a group lymph nodes were sorted separately. All specimens and sorted lymph nodes were fixed in 10% formaldehyde solution within 1 h and sent to the pathology department for testing (Kaida S, Murata S, Miyake T, et al 2022; Liang, H 2019; Ojima T, Nakamura M, Nakamori M, et al. 2018; Gastric Cancer Professional Committee et al. 2022).

### Various tube placement and abdominal closure techniques

① Placement of abdominal drainage tube: Try to attract clean abdominal and pelvic irrigation water before placing the drainage tube; when attracting, a piece of gauze can be placed in front of the suction device, attracting through the gauze will prevent the intestinal tube or tract from blocking the suction device. The drainage tube is usually placed at the gastro-intestinal anastomosis, esophagus-jejunum anastomosis, and duodenal stump, taking into account the spleen fossa and liver and kidney interstitial space, but not in the pelvis. After placement of the drainage tube, the drainage tube can be fixed by suturing at the skin of the drainage tube, and the sutures should be sutured as tightly as possible to the skin outlet to reduce leakage and prevent the drainage tube from dislodging, and the drainage device should be attached (Weindelmayer et al. 2021).

② Gastric tube and jejunal nutrition tube placement: Total gastrectomy patients can be placed without a gastric tube; distal gastrectomy and proximal gastrectomy patients can be placed with a gastric tube to 10 cm distal to the gastrointestinal or esophageal intestinal anastomosis; jejunal nutrition tube is suitable for patients after all types of gastric surgery and is mostly placed 10 cm distal to the intestinal anastomosis; the above tubes are placed at the end of the intraluminal anastomosis or when the small incision is open for anastomosis. A small amount of saline is injected into the tube while entering, which can facilitate the tube entry. After entering into the designated position, the gastric tube and jejunal nutrition tube are properly fixed with adhesive tape, and it is recommended that the 2 tubes be placed in the same nostril, which can keep the other nostril open for breathing (Wang et al. 2022a; Dann et al. 2015).

③ Suturing and dressing techniques for abdominal wall incision: After the surgery, try to drain the gas in the abdominal cavity. Eight millimeters and below perforation holes can be sutured only the subcutaneous layer and skin, 12-mm perforation holes and specimen removal incision all layers of the abdominal wall need to be completely sutured to prevent incisional hernia from occurring. In case of difficulty in suturing the puncture hole, a disposable puncture hole suture can be used, or the handle of the forceps can be inserted backwards into the puncture hole along the direction of the skin incision, and the whole layer of the abdominal wall can be picked up by tilting the forceps to one side of the incision, and after checking that there is no error, the peritoneum and abdominal fascia can be sutured by clamping the tissue on that side with vascular forceps, and the whole layer of the abdominal wall can be picked up by tilting the forceps to the opposite side of the incision in the same way and continuing to be sutured, and the 8-string suture can be sutured after A square knot is tied and fixed, and the puncture hole is checked for complete suturing, if not complete the previous suture can be lifted and sutured again. During the suturing process, wipe the incision as clean as possible to ensure exact suturing. It is recommended to suture in layers, and to rinse the incision twice with dilute iodine saline when preparing to suture the subcutaneous and skin layers. In obese patients, drainage skin pieces or thin drainage tubes can be placed under the skin to promote the drainage of subcutaneous blood and exudate. The surgical incision can be covered with iodophor gauze and then fixed with a dressing, and the abdomen can be wrapped with a lap band to reduce abdominal wall pain during activity or coughing (Lesch et al. 2022).

### Intraoperative complication prevention and control experience

① Pneumoperitoneum-related complications: Intraoperative pneumoperitoneum pressure should be maintained at 12–14 mmHg, with a flow rate of about 40 L/min. When entering the puncturer, the whole layer of the abdominal wall should be penetrated at once before inflation to avoid gas entering the outer layer of the peritoneum. The abdominal wall incision and the puncturer should be fitted as closely as possible to avoid subcutaneous emphysema. When separating the esophageal and pleural adhesions by total gastrectomy, the esophageal plasma membrane layer should be tightly adhered to keep the pleura intact as much as possible to avoid pneumothorax. The additional muscarinic drugs should be used to maintain a good muscarinic state during the operation to minimize the operation time. Before closing the abdomen after surgery, try to gently squeeze the abdomen to expel the residual carbon dioxide gas in the abdominal cavity and reduce the postoperative discomfort of the patient’s shoulder and back (Watrowski et al. 2021).

② Intraoperative vascular injury: Pay attention to protect the inferior vena cava, abdominal aorta, abdominal trunk, splenic artery, portal vein, common hepatic artery, intrinsic hepatic artery, GDA, and other vessels intraoperatively. When the tumor invades the above vessels, pay attention to the intermediate open or palliative surgery, and if necessary, artificial vascular replacement can be done. Prepare for blood transfusion before surgery. If there is more bleeding during surgery, contact blood bank urgently for blood transfusion and increase the amount of rehydration to maintain blood volume. Try to ligate two hemostatic clips at the preserved end of the vessel when thicker vessels are encountered during surgery. Try not to separate the veins too thinly during surgery to prevent rupture of the vessels or dislodgement of the vessel clips when too little tissue is clamped. Injuries to the liver, spleen, and other parenchymal organs can be filled with hemostatic gauze locally to promote hemostasis. Bleeding from the intestinal canal, mesentery, and vascular dissection can be stopped by local sutures. For bleeding from the surface of the pancreas and other organs, wet gauze can be used to cover the bleeding site and electrocoagulation hook can be used to stop the bleeding through the gauze, which can stop the bleeding without damaging too much pancreatic tissue. If a small amount of blood is oozing from the surgical wound, it can be cleaned up with gauze wipes or a suction device, and the surgical field can be rinsed with water when it is not clear due to blood oozing before continuing the operation. If intraoperative bleeding is difficult to control, an assistant should hold the gauze with intestinal forceps to press the bleeding site and immediately transit the open abdomen to stop the bleeding (Watrowski et al. 2021; England et al. 2020).

③ Intraoperative adjacent organ injury: Intraoperative access to the puncturer may damage the intestinal canal, mesentery, omentum, and liver, etc., and suspension of the liver may damage the diaphragm, pericardium, and liver. The transverse colon, transverse mesentery, and gallbladder may be damaged when removing the greater omentum. The spleen may be damaged when clearing the lymph nodes of the splenic portal, the liver may be damaged when clearing the lymph nodes of the gastric lesser curvature test, and the pancreas and duodenum may be damaged when clearing the posterior wall of the stomach and the pylorus. For tumor invasion of surrounding organs, the surgical risk and patient’s prognosis should be evaluated, and combined organ resection should be performed if necessary. Intraoperative damage to intestinal canal, mesentery, omentum, omentum, liver, colon, spleen, and other organs should be treated with organ repair and hemostasis. If necessary, intraoperative consultation with specialists in the relevant specialties should be requested to assist in the operation. For organs that need to be resected when they have become severely ischemic and cannot be repaired, the informed consent for surgery needs to be indicated in the informed consent form and written consent from the patient’s family before resection (Watrowski et al. 2021; Velilla et al. 2018).

### Postoperative complication management experience

① Abdominal bleeding: Abdominal bleeding in the first 3 days after surgery is mostly due to incomplete intraoperative vascular electrocoagulation to stop bleeding or poorly secured and dislodged vascular clamps, postoperative blood pressure rebound, too rough lifting and placing movements, abdominal exertion, coughing, malignancy, vomiting, etc. Resulting in vascular rupture or tissue bleeding: abdominal bleeding after 3 days is mostly due to ruptured bleeding from pancreatic leakage, intestinal leakage, abdominal infection, etc. Resulting in erosion of the vascular wall in the surgical area: In addition, patients with low platelets and poor coagulation function before surgery, high intraoperative bleeding, long operation time, and premature and excessive use of anticoagulants after surgery can also lead to bleeding. When abdominal bleeding occurs, the drainage tube should be kept open, and abdominal bleeding should be judged by observing the drainage flow and the hemoglobin level in the blood routine. Patients may experience irritability, abdominal discomfort, and nausea during bleeding. As the bleeding volume increases, the patient will show a decrease in blood pressure and an increase in heart rate. Give immediate symptomatic treatment such as cardiac monitoring, oxygenation, infusion of hemostatic drugs, fluid replacement, and blood transfusion, and recheck blood routine and coagulation function. When the bleeding volume is greater than 100 ml/h, open abdominal exploration should be performed immediately under general anesthesia for hemostasis, and selective vascular occlusion under local anesthesia for hemostasis can also be performed under radiological intervention (Watrowski et al. 2021; Zizzo et al. 2022; Chen et al. 2022a).

② Treatment of incisional bleeding: If active bleeding is found in the skin and subcutis of the incision after surgery, the posterior lap band can be covered with a gauze pad and pressure bandaged first, and bleeding can still not stop by performing 8-string suturing of the skin and subcutaneous tissue at the bleeding site under local anesthesia. If the hemostasis is poor, the skin and subcutaneous layer are opened, and the skin is re-sutured after ligation to stop the bleeding. The sutures should not be too deep to prevent suturing to the intestinal canal and other organs. If bleeding from the puncture hole into the abdominal cavity is ineffective, body sutures can be performed again for laparoscopy or complete opening of the incision for exploration and exact hemostasis before closing the abdomen (Zhu et al. 2020).

③ Anastomotic bleeding: Anastomotic bleeding is mostly discharged from the digestive tract in the form of vomiting blood and blood in the stool. Small amounts of bleeding can be clearly diagnosed by gastric fluid and fecal occult blood tests, medium amounts of bleeding can have coffee-like gastric fluid or black stool, and large amounts of bleeding can have bright red gastric fluid or dark red blood in the stool. A small amount may also enter the abdominal cavity in the form of abdominal bleeding. Patients may experience irritability, abdominal discomfort, and nausea during bleeding. As the bleeding increases, the patient may show a decrease in blood pressure and an increased heart rate. Postoperative anastomotic bleeding can be observed by placing a gastrointestinal decompression tube to drain the bleeding, or by administering medication via a gastric tube. In case of a small amount of bleeding, norepinephrine mixed with ice saline, Yunnan Baiyao, and aluminum magnesium plus suspension can be injected in parts and the gastric tube can be closed for 1 h and then opened to observe the bleeding, and if necessary, the drug can be given repeatedly. At the same time, give cardiac monitoring, oxygen, infusion of hemostatic drugs, rehydration, blood transfusion and other symptomatic treatment, and recheck the blood routine and coagulation function. When the bleeding volume is greater than 100 ml/h, the patient’s vital signs and the number of hemoglobin and platelets allow for immediate hemostasis by endoscopic sclerotherapy under surface anesthesia, hemostatic clips, etc. Selective vascular occlusion under radiological intervention can be performed under local anesthesia, or open surgery under general anesthesia for anastomotic hemostasis and reconstruction (Park et al. 2020).

④ Anastomotic fistula: Anastomotic leak mostly occurs after anastomotic bleeding and is mostly due to local tissue ischemia. After the occurrence of a leak, complete fasting of food and water should be performed, and a gastric tube should be left in place at the anastomosis to continuously aspirate digestive fluid from the lumen outward. The abdominal drainage tube should be kept open, and if necessary, ultrasound-guided laparotomy should be performed to increase the number of drains or replace them with double cannulae. Growth inhibitors are given to pump in to reduce digestive fluid secretion and antibiotics to prevent infection. If the anastomotic leak does not heal after conservative treatment, anastomotic repair and reconstruction can be performed by placing a laminated stent under gastroscopy or by open surgery under general anesthesia. During the treatment period, parenteral nutrition should be enhanced, and enteral nutrition should be supported by a jejunal nutrition tube, while albumin should be administered to promote the healing of the leak (Trapani et al. 2020).

⑤ Leakage of duodenal stump: Leakage of duodenal stump mostly occurs after anemia, hypoproteinemia, anastomotic bleeding, intestinal obstruction, or intraoperative duodenal freeing for too long, mostly due to local tissue ischemia. After leakage occurs, complete water fasting, continuous gastrointestinal decompression by gastric tube, and growth inhibitor pumping should be given to reduce the secretion of digestive juices and antibiotics to prevent infection. The abdominal drainage tube should be kept open, and if necessary, ultrasound-guided laparotomy placement should be performed to increase the number of drainage tubes or replaced with double cannulae. If conservative treatment is ineffective, open surgery under general anesthesia may be performed for stump repair and reconstruction. During the treatment period, parenteral nutrition should be enhanced, and enteral nutrition should be supported with a jejunal nutrition tube, while albumin should be administered to promote healing of the leak (Zizzo et al. 2019).

⑥ Pancreatic leak: Pancreatic leak mostly occurs due to injury to the pancreas at the head of the pancreas separating the right vessels of the gastric omentum and at the tail of the pancreas clearing the lymph nodes around the splenic hilum. After leakage occurs, complete fasting of food and water, continuous gastrointestinal decompression by gastric tube and octreotide pumping should be given to reduce digestive fluid secretion. The abdominal drainage tube should be kept open, and if necessary, ultrasound-guided laparotomy should be performed to increase the number of drains or replace them with double cannulae. During the treatment period, parenteral nutrition should be enhanced, and enteral nutrition support should be provided under a jejunal nutrition tube, while albumin should be input to promote the healing of the injury (Wu et al. 2022).

⑦ Celiac leakage: Which mostly occurs when the lymphatic vessels are not clamped during lymph node dissection on the side of the gastric lesser curvature. After leakage, fatty milk infusion and fatty food intake should be stopped, the abdominal drainage tube should be kept open, and parenteral nutrition support should be strengthened and enteral nutrition should be reduced appropriately during treatment, while albumin should be infused to promote healing of the injury. If conservative treatment is ineffective, fibrin glue treatment is feasible, and laparoscopic exploration is performed to clip the lymphatic vessels at the leak or to perform abdominal venous shunts (Kong et al. 2022; Sakamoto et al. 2022; Mahmoodzadeh et al. 2021).

⑧ Anastomotic stenosis: Anastomotic stenosis is mostly caused by too small anastomosis or scar growth, and tubular anastomosis causes anastomotic stenosis more often. Balloon dilatation or stent placement can be performed under gastroscopy, and if the symptoms are still not improved, anastomotic reconstruction can be performed under general anesthesia with open or laparoscopic surgery (Manaka et al. 2022).

⑨ Abdominal and pelvic infections: Abdominal and pelvic infections mostly occur after anastomotic leak, duodenal stump leak, pancreatic leak, celiac leak, etc. or small bowel and colon leak caused by intraoperative injury or local ischemic necrosis. Or secondary infections due to acute inflammatory episodes of the appendix and gallbladder. After the occurrence of infection, the abdominal drainage tube should be kept open, and if necessary, ultrasound-guided laparotomy placement should be performed to increase the number of drains or replaced with double-cannula flushing. According to the results of bacterial culture and drug sensitivity of drainage fluid, two or more strong antibiotics should be combined to enhance anti-infection treatment. During this period, parenteral nutrition should be intensified and enteral nutrition support under jejunal nutrition tube should be performed (Ojima et al. 2022).

⑩ Abdominal and pelvic fluid: Residual abdominal and pelvic fluid is mostly due to incomplete intraoperative flushing fluid suction, low postoperative patient activity, poor placement of drainage tube, local folding of drainage tube, or blockage of blood clots. The abdominal ultrasound can be reviewed in the postoperative period to detect ascites, and if the amount of fluid is large, the drainage tube can be withdrawn 2–3 cm outside the abdominal cavity during incisional dressing change, and the drainage tube can be squeezed to increase the amount of movement to promote drainage fluid discharge. If the fluid is still not drained, ultrasound-guided laparoscopic aspiration or drainage is feasible. If it is still ineffective, endoscopic drainage through the wall is feasible. If the amount of fluid is small, the abdominal drainage tube can be removed and fixed with thick gauze, and appropriate exercise can make it flow out or absorb on its own (Donatelli et al. 2018).

⑪ Gastroparesis: The risk of gastroparesis is increased with a residual stomach greater than 1/3 after distal gastrectomy, which may be associated with lack of blood supply to the gastric wall and vagus nerve dissection, hypoproteinemia, and local inflammation. The diagnosis can be further clarified by performing upper gastrointestinal imaging with oral cotrimoxazole. If there is no change in the intra-gastric contrast within half an hour and the gastric tube can aspirate more than 400 ml of gastric juice per day, the diagnosis can be made. Fasting with water, continuous gastrointestinal decompression, parenteral nutritional support, and downward mobility are recommended. If necessary, erythromycin drip is given to promote gastric motility (Mukoyama et al. 2022).

⑫ Intestinal obstruction: Intestinal obstruction is mostly caused by postoperative abdominal inflammation, edema, bed rest, and pain medication. After the occurrence of intestinal obstruction, complete water fasting, continuous gastrointestinal decompression by gastric tube, and octreotide pumping should be given to reduce the secretion of digestive juices. Treatment such as enema can be given appropriately. If the treatment is ineffective after conservative treatment, open abdominal exploration can be performed under general anesthesia. During the treatment period, parenteral nutrition should be enhanced, and enteral nutrition support under jejunal nutrition tube should be performed in parallel, while albumin should be input to promote the healing of the injury (Li et al. 2022a).

⑬ Surgical incision infection: After surgery, the incision has a small amount of gray-white purulent fluid outflow, consider local infection of the incision, can be appropriately expanded after the incision with hydrogen peroxide and saline repeatedly rinse, to clear rinse solution in the incision after stuffing iodine voltaic gauze strip drainage. If the incision has a large amount of gray-white purulent fluid with foul odor, consider that all the incision is infected, first take part of the pus for bacterial culture, then dismantle the skin of the incision and all the sutures under the skin, rinse repeatedly with hydrogen peroxide, iodophor water, and a large amount of saline until the rinsing fluid is clear, then wipe repeatedly with dry cotton balls or gauze to remove the necrotic material in the incision, insert iodophor gauze strips in the incision, and cover with thick gauze to fix it. The incision should be flushed and the iodophor gauze strips should be replaced at each change until the secretion in the incision is gradually reduced and the redness and swelling subside. Those with fever and elevated white blood cells may be treated with systemic antibiotic infusion for 5–7 days depending on the bacterial culture results. When the incision gradually becomes shallow and small, the number of gauze strips filled in the incision is reduced, and after fresh granulation tissue grows, recombinant human epidermal growth factor solution or compound comfrey oil can be sprayed locally in the incision to promote the healing of the incision, and the skin on both sides of the incision can be brought together in the middle by using adhesive tape to stick the incision vertically. At this time, the interval between drug changes was changed to 2–3 days, and the lap band was wrapped tightly after the drug change to prevent poor healing of the incision scar causing incisional hernia. One-stage resuturing of infected incisions is generally not recommended, and elective scar excision plastic surgery is performed when necessary after complete healing of the incision (Zhao et al. 2022; Han et al. 2020).

### Postoperative patient management considerations

① Anesthesia recovery and monitoring: Patients are sent to the anesthesia recovery room (PICU) after leaving the operating room to observe the awakening and whether they can be extubated. If the intraoperative bleeding is large, the patient’s basic condition is poor, anesthesia recovery is slow, and they cannot be extubated smoothly, they can be temporarily transitioned in the ICU and then transferred back to the general ward to continue treatment when their condition improves. It is recommended that patients be given continuous integrated cardiac monitoring and oxygenation for 2–4 L/min for at least 24 h after surgery, and the level of care can be reduced after all vital signs are stable (Jahangir et al. 2020).

② Postoperative fluid management and nutritional support: Postoperative routine rechecking of arterial blood gas, blood routine, biochemistry, coagulation function, and drainage fluid amylase every 1–2 days, timely observation of the patient’s postoperative condition, maintenance of water-electrolyte acid–base balance, and timely management of any abnormalities. Parenteral nutrition support and jejunal nutrition tube infusion of glucose sodium chloride solution can be given to patients 1 day after surgery, and enteral nutrition support can be given 2 days after surgery. Postoperatively, fluid volume, caloric volume, nitrogen volume, electrolytes, vitamins, and other intakes are calculated comprehensively according to the patient’s weight, nutritional status, and drainage volume to meet the patient’s nutritional needs as comprehensively as possible (Lahoud et al. 2019).

③ Postoperative antibiotic management: Continue to give broad-spectrum antibiotic treatment for 3–5 days after surgery, routinely recheck blood routine, rapid C-reactive protein, calcitoninogen, and sedimentation every 1–2 days, and parallel abdominal drainage fluid, urine or sputum bacterial culture + drug sensitivity test, measure the patient’s body temperature on time, and promptly deal with any abnormalities (Yao et al. 2022).

④ Postoperative dietary management: Postoperative diet can be gradually transitioned from drinking water, liquid diet, semi-liquid diet, soft food to general diet, and follow the dietary principles of small amount, multiple times, and gradual increase in quantity. If abdominal pain, abdominal distension, nausea, vomiting, indigestion, and other symptoms appear after eating, the amount or number of diet should be reduced, observe whether the symptoms can be relieved after appropriate exercise, pay attention to abdominal warmth which can promote intestinal peristalsis, if the above symptoms cannot be relieved, give symptomatic treatment, take hypertonic effect which can promote gastrointestinal peristalsis to reduce nausea and vomiting symptoms; if necessary, leave a gastrointestinal decompression tube, negative pressure suction to relieve gastrointestinal symptoms. The diet should be soft and easy to digest, raw, cold, greasy, hard, and irritating foods should be avoided, and the diet should consist balanced nutrition and variety (Jang and Jeong 2021).

⑤ Postoperative exercise management: Early bed activity should be encouraged. Patients should be encouraged to get out of bed early, turn and move their limbs in the hospital bed with the assistance of nurses the night after surgery, walk around the bedside and ward 1 day after surgery, and perform basic normal activities 2 days after surgery. The accelerated rehabilitation surgical concept emphasizes early bed activity because it can increase bowel movement and lung activity to improve resistance to disease and speed up physical recovery, as well as accelerate blood circulation at the incision site to promote incision healing and venous reflux in the lower extremities and prevent the formation of postoperative deep vein thrombosis to reduce the occurrence of postoperative complications (Pang et al. 2022).

⑥ Postoperative pulmonary function protection: On the day of surgery, the patient was placed in a flat position with the head turned to one side to keep the airway open. One day after surgery, the patient is given a semi-recumbent position and may be given nebulized inhalation, back patting or mechanically assisted sputum excretion, and aspiration with a suction tube if necessary (Ong et al. 2022).

⑦ Postoperative VTE prevention experience: On the day after surgery, according to the patient’s specific situation, hemostatic drugs may be given temporarily once. Routine coagulation tests should be done 24 h after surgery, and subcutaneous injection of low-molecular heparin calcium may be given according to the color of the drainage fluid to prevent deep vein thrombosis and pulmonary artery embolism in the lower extremities with anticoagulation therapy. Patients who fail to get out of bed in the first few days after surgery can continue to wear elastic stockings, move their limbs, and massage both lower limbs, and hospitals with conditions can give pneumatic massage treatment and promote blood circulation by turning and patting the back appropriately (Wang et al. 2020b).

⑧ Postoperative analgesic management experience: Patients can be given an intravenous indwelling analgesic pump for analgesia within 24–48 h after surgery, followed by short-acting analgesic treatment. Intravenous infusion of analgesics can also be given for symptomatic treatment, or skin paste of fentanyl transdermal patch for pain relief, and intramuscular injection of morphine-based analgesics if necessary (Zhang et al. 2018).

⑨ Medication change technique for surgical incision: Change the medication for surgical incision every 2–3 days, and pull out the drainage skin piece or subcutaneous drainage tube when there is no exudate from the incision. If the incision is red, swollen, hot, painful, purulent exudate and other manifestations need to promptly explore the incision when changing the medication, you can first use lidocaine injection local anesthesia and then use sterile forceps to insert and expand the incision 0.5–1 cm from the secretion outflow, and slightly squeeze to check the incision. If the incision has a small amount of bright red blood outflow, consider the incision normal, local iodophor disinfection, and gauze wrapping fixed. If the incision has a small amount of light red or dark red bloody fluid outflow, consider the incision fluid and blood accumulation which can be localized by inserting gauze strips for drainage. If there is a small amount of yellowish grease-like liquid out of the incision, consider subcutaneous fat liquefaction of the incision which can be drained by locally inserting gauze strips. The incision can be treated with red light irradiation to promote recovery (Blumenthaler et al. 2021).

⑩ The urinary catheter can be removed: Postoperatively, the urine volume is observed and recorded daily, and routine urinalysis is performed if necessary, and the urinary catheter can be closed regularly on the first postoperative day to exercise bladder function. Oral tamsulosin can be given to those who have difficulty in urination after removal of the tube, and catheterization can be repeated in severe cases (Ma et al. 2021).

⑪ Gastrostomy tube removal: Postoperatively, the color of the drainage fluid was observed and the drainage flow was recorded every day, and if necessary, the latent blood test of the drainage fluid was performed. The gastric tube can be removed when there is no nausea or vomiting after eating a liquid diet after surgery (Ma et al. 2021).

⑫ Jejunal nutrition tube removal: The jejunal nutrition tube can be removed at a later date according to the patient’s eating condition, and the placement time can be extended appropriately when the preoperative nutritional status is relatively poor and postoperative abdominal infection, intestinal obstruction, gastroparesis, anastomotic leakage, anastomotic bleeding, etc. occur (Ma et al. 2021).

⑬ Abdominal drainage tube removal: When the abdominal drainage tube drainage fluid is less than 10 ml for 2 consecutive days, the drainage fluid amylase is normal, and there is no abnormality after eating a liquid diet, the abdominal drainage tube can be removed, but it is better to do an ultrasonic ascites probe before removal to ensure that no fluid remains in the abdominal cavity. When removing the drainage tube, cut the drainage tube fixation line, loosen the skin next to the drainage tube with forceps, let the patient take a deep breath first, and remove the drainage tube when the patient slowly breathes out. After removal, disinfect the drainage hole, use adhesive tape to vertically adhere the skin on both sides of the drainage hole to the middle, and then cover with thick gauze wrap to fix it to prevent local leakage. If the patient has more exudate at the drainage hole, the drainage hole can be sutured under local anesthesia and the stitches can be removed after it has healed (Ma et al. 2021).

### Experience in the management of common postoperative abnormalities

① Cardiac arrhythmia: Patients’ postoperative heart rate will be accelerated due to surgical and anesthetic trauma and pain, which may induce arrhythmia. When the heart rate increases, patients need to be screened for common causes such as acute pain, anemia, atrial fibrillation attack, myocardial infarction, and heart failure. Promptly perform electrocardiogram, cardiac ultrasound, cardiac enzymes, myocardial markers, and other tests, and if necessary, coronary CTA or coronary angiography, cardiac radiofrequency ablation. According to the test results, invite relevant departments to consult and assist in diagnosis and treatment. If the heart rate is slowed, the sinus node and atrioventricular conduction function should be investigated for abnormalities, and atropine test, esophageal pacing, and temporary artificial pacemaker placement should be given if necessary (Rühlmann et al. 2022).

② Breathing difficulties: It may be due to various factors such as pain, anemia, abdominal infection, pulmonary infection, pulmonary atelectasis, pleural effusion, cardiac insufficiency, pulmonary embolism, and pulmonary insufficiency. Test finger pulse oxygen saturation and arterial blood gas, perform chest X-ray and CT examination of chest and abdomen, and if necessary, perform pulmonary CTA or pulmonary arteriography. Give symptomatic treatment according to the examination results and transfer to ICU for invasive ventilator-assisted ventilation with tracheal intubation in severe cases (Wang et al. 2022b).

③ Abnormal blood pressure: Examine whether the elevated blood pressure is due to abdominal pain, malignancy, vomiting, etc., if there is no such cause. Short-term elevation can be lowered with sublingual captopril tablets, higher with oral nifedipine tablets, and ineffective with controlled hypotension with intravenous pumping of uradil. If the blood pressure decreases, investigate whether it is due to low rehydration, poor heart function, high drainage, anemia, abdominal bleeding, etc., and give symptomatic treatment. If it is still low, measures such as massive blood transfusion, rehydration, and dopamine pumping can be given to raise the blood pressure (Sun et al. 2018).

④ Abnormal blood glucose: When blood glucose is elevated, check whether there is a history of diabetes or the liquid is being infused with glucose, such as infusion of nutritional fluid, check whether the amount of insulin in the liquid is sufficient, and the insufficient amount can be appropriately supplemented, and retest the blood glucose after half an hour until it is reduced to a controllable range. When blood glucose is reduced, check whether it is caused by fasting, insufficient energy rehydration in the fluid or excessive insulin addition, etc. If it is too low, 10% glucose injection of 250 ml can be given as an IV, and blood glucose needs to be retested after half an hour until it rises to the appropriate range (Sun et al. 2018).

⑤ Fever: Postoperative fever may be due to tissue absorption of heat, and the general body temperature is lower than 38.0 °C, which can be physically cooled by giving ice water wipes and other methods. Above 38.5 °C needs to be given medication to cool down, such as Chai Hu injection + analgesic injection or indomethacin bolus in the anus, and ibuprofen is not recommended for oral administration. If the patient sweats a lot during the cooling process, increase the amount of rehydration fluid. And continue to monitor body temperature and investigate for deep vein catheter infection, abdominal infection, anastomotic leak, pulmonary infection, and urinary tract infection. In the case of hyperthermia, blood can be drawn simultaneously for bacterial culture, and if necessary, chest X-ray, CT or abdominal ultrasonography, bacterial culture of deep venous catheter, abdominal drainage fluid, sputum, and urine (Zheng et al. 2019).

⑥ Oliguria: When the 24-h urine volume is less than 300 ml, the cause of oliguria needs to be investigated, such as renal insufficiency and insufficient rehydration. Check renal function, blood pressure, and other indicators, and those who have insufficient rehydration fluid can be given increased rehydration volume, appropriate diuretics are given to observe whether the urine volume increases and whether urea nitrogen, creatinine, and blood potassium decrease, and if all improve, continue treatment; otherwise, give hemodialysis (Kubicki et al. 2018).

⑦ Insomnia: Postoperative noisy environment, abdominal discomfort and sleep reversal can lead to insomnia at night, by reducing the patient’s daytime sleep time at night before sleep can be given clonidine to promote sleep and maintain deep sleep with eszopiclone tablets (Zhu et al. 2021).

⑧ Anxiety, depression and delirium: Postoperative patients can suffer from anxiety, depression, and delirium due to anesthesia, surgical trauma, pain, drains, catheters, and insomnia. Patients are given psychological counseling and sedative medications such as risperidone, haloperidol, and quetiapine are given appropriately, and neurological consultation is requested to assist in the diagnosis and treatment if necessary (He et al. 2022).

⑨ Nausea and vomiting: Patients may experience nausea and vomiting on the postoperative day due to the reaction to anesthetic drugs and can be treated symptomatically with gastrofacial or other antiemetic medications. If symptoms such as nausea and vomiting occur after postoperative feeding, anastomotic obstruction, output intestinal collateral obstruction or gastroparesis should be alerted, and oral cotrimoxazole can be administered to perform upper gastrointestinal imaging to further clarify the diagnosis. The original solution of pantothenic glucosamine can promote gastrointestinal motility and reduce nausea and vomiting due to its hypertonic effect. Acupuncture or acupuncture point treatment can also be given (Chen et al. 2022b).

⑩ No anal defecation: Patients may have no anal defecation after surgery due to the use of anesthetic drugs and painkillers, bed rest, abdominal edema, adhesions, abdominal pain, and other reasons, first pay attention to abdominal warmth to promote intestinal peristalsis, abdominal microwave therapy can be given, laxatives or lactulose oral symptomatic treatment can also be given, and if the symptoms do not relieve, enema treatment can be given, acupuncture or acupuncture point treatment can also be given (Li et al. 2022b).

### Enhanced recovery after surgery (ERAS) management experience

The ERAS model can be used for the perioperative management of patients with relatively good physical underlying conditions and under 70 years of age to promote rapid recovery. The principles of perioperative ERAS management include detailed preoperative assessment, adequate cardiopulmonary exercise, effective control of underlying diseases, precise surgical operation, shortened operative time, refined postoperative management, individualized rehabilitation plan, and close multidisciplinary cooperation (Jeong and Kim 2019).

① Preoperative interventions: Including preoperative education, assessment of nutritional status and intensive nutritional therapy, preoperative bowel preparation, fasting with oral carbohydrate (500–1000 ml of sugar saline given orally 4 h before surgery (caution in patients with pyloric and cardia obstruction)), prophylactic use of antimicrobial drugs, control of underlying diseases and reserve function training, and DVT prevention (D'Ugo et al. 2020).

② Intraoperative interventions: Including intraoperative insulation, drainage, catheter optimization, anesthesia protocol, intraoperative DVT prophylaxis, and minimally invasive surgery (Wee et al. 2019).

③ Postoperative interventions: Postoperative accelerated recovery management experience includes the use of multimodal analgesic protocols (pain pumps can be left in place and long-acting analgesics are used for regular intravenous infusion), minimizing the placement and early removal of all types of catheters (deep vein placement, gastric tubes, nutrition tubes, and abdominal drains can be left in place), and resuming oral feeding as soon as possible to promote gastrointestinal recovery (oral feeding as early as possible after surgery, from the first postoperative day onwards from water (fluid diet, semi-liquid diet, soft food, and general diet) and early bedtime activities (after complete awakening from anesthesia on the postoperative day, bedtime activities can be performed with the assistance of medical staff) (Yang et al. 2020b; Wang et al. 2022c).

### Discharge criteria and postoperative follow-up experience

① Discharge criteria: The patient can eat semi-liquid or soft food through the mouth, the amount of food is about 200 ml/time, there is no obvious choking feeling when eating, and there is no obvious malignancy, vomiting, abdominal pain, and other discomfort after eating. The anus can vent and defecate normally without obvious black stool. All kinds of drainage devices and deep venous catheters were basically removed. The incision was healed at grade A/B. No complications such as fever, lung infection, anastomotic fistula, and stenosis. Blood routine, urine routine, biochemistry, coagulation function, and other indicators tend to be normal, abdominal ultrasound and chest X-ray examination did not show obvious fluid accumulation and lung exudation. If the above criteria were not met, it was recommended to extend the hospital stay or transfer to a local hospital for treatment (Yu et al. 2021).

② Postoperative follow-up and review: Patients with gastric cancer can be followed up and reviewed in the local hospital or outpatient clinic of the operating hospital after surgery, which is recommended once a month within 6 months after surgery, once every 3 months after 6 months, once every 6 months after 1 year, and once a year after 2 years until the end of the 5-year postoperative follow-up. Each follow-up visit and review includes measuring weight change, assessing nutritional status, checking incision healing, asking about diet, sleep, urine and stool, etc. The routine blood, urine, stool, biochemistry, tumor markers, coagulation function, etc., abdominal ultrasound, chest X-ray, and enhanced CT examination if necessary. Electronic gastroscopy is recommended to be reviewed once every 6 months. Patients who require chemotherapy during the follow-up period should also complete chemotherapy according to an individualized protocol (Qiu et al. 2022).

## Discussion

This paper summarizes 20 experiences of perioperative clinical management of robotic gastric cancer surgery based on the Chinese Guidelines for Robotic Gastric Cancer Surgery combined with the experiences summarized in actual clinical work, and supplements and expands the relevant details based on other surgical guidelines, consensus and single-center experience, which can make the guidelines better guide clinical practice. ① Advantages: This management experience takes clinical guidelines as the theoretical guidance and clinical practice as the test standard, so that theory can be combined with practice to solve the problems encountered in actual clinical work. ② Limitations: Our experience mainly comes from single-center clinical work and needs to be fully validated and supplemented in multi-center, large sample and high-quality clinical RCT studies in the future. Details determine success or failure, good surgical outcomes and postoperative recovery cannot be achieved without every aspect of the perioperative period and may every surgical patient be safely discharged from the hospital.

## Conclusions

We hope our experience can bring some clinical reference value to the front-line clinicians in the treatment of robotic gastric cancer surgery.

## Data Availability

We certify that we have participated sufficiently in the work to take public responsibility for the appropriateness of the experimental design and method, and the collection, analysis, and interpretation of the data. All the data are valid and available.
